# A Course of Clinical Lectures, Delivered at the Hôtel-Dieu, Paris, for the Session 1842-’43

**Published:** 1843-03-04

**Authors:** A. F. Chomel

**Affiliations:** Paris


### THE MEDICAL EXAMINER,

### And Retrospect of the Medical Sciences.

Vol. VI.] PHILADELPHIA, SATURDAY, MARCH 4, 1843. [No. 4.

### A COURSE OF CLINICAL LECTURES,

*Delivered at the Hôtel Dieu, Paris, for the Session 1842–'43.*

BY A. F. CHOMEL, M. D.

### LECTURE III.

Case I.—*Phthisis, complicated with ascites and œdema of the lower extremities—Anatomical lesions corresponding to these phenomena.*

Gentlemen:—At no. 33 of the Salle St. Bernard I have called your attention several times to a woman with all the symptoms of advanced pthisis. I shall not now dwell upon the symptoms of this affection, which offer nothing in this instance worthy of note; but call your attention to the complications which existed in this patient, and which are rare in this disease—the œdematous swelling of the lower extremities, with ascites. Now you meet very rarely with these phenomena in phthisical patients, and hence it is interesting to inquire into the causes of these symptoms. Let us first examine into the ordinary causes of ascites.

Ascites depends on some material lesion of some one of the abdominal organs rather than upon any general cause, as obstruction in the venous circulation, or a state of general anemia. The most common cause of peritoneal ascites is an organic affection of the liver, as cirrhosis, for example. Now this affection rarely exists in tuberculous patients. It is true that tuberculous disease itself may produce serous effusion in the peritoneal cavity; the tubercles exist in the mesentery becoming developed, compress the vessels which lie along the vertebral column, and by impeding the abdominal circulation, produce ascites. There is also an affection of the liver found frequently in phthisis, and which can exercise a certain influence in the production of the phenomena we are analyzing—I mean the *fatty liver.* The ascites and œdema in this case may have been due to the existence of mesenteric tubercles compressing the large abdominal vessels, or to the fatty liver peculiar to phthisis, or to both these causes united. In regard to the other alteration of the liver, called cirrhosis, we might also suspect its existence, but it is extremely rare in phthisical patients. There was also in this patient absence of diarrhœa, another peculiarity very rare in advanced phthisis. Now, all these symptoms are worthy of your attention. Having now briefly recapitulated the phenomena, I shall proceed to the explication of the precise cause of the rare morbid phenomena we have noticed, which the autopsic examination has enabled us to do, the patient having died since our last visit. Among the possible causes of these two results, I mentioned the compression of the vessels by tubercles in the mesentery. At the autopsy we found the femoral veins, and the clot of recent organization; it was probably formed about a week before death. This lesion explains only the anasarca. For the ascites we supposed the probability of the existence of the fatty degeneration of the liver, or the possibility of cirrhosis, this latter, however, seeming to us as very doubtful. On examination after death we found the liver firm, consistent, but small, shrivelled, and a little rough on the surface; in cutting it we found the tissue compact, less thick, but more resisting than ordinary, and grating under the scalpel; an attentive examination showed us on the surface several yellow points, and about the biliary ducts the tissue was of a yellow colour, which is characteristic of cirrhosis. There was the commencement of this affection; and it is very probable that it was the real cause of the ascites observed during life. It is very true that cirrhosis was very little advanced in this patient, but it was sufficiently characterized to induce us to consider it as the true and only cause of the ascites. We found nothing in the veins, and besides any alteration in them does not in general produce ascites till a remoter period. We found, too, in this patient some hypertrophy of the right ventricle of the heart, which no doubt aided to produce the œdema of the lower extremities. The absence of diarrhœa, which is generally so common a symptom in the last stage of phthisis, can be explained by the non-existence of those intestinal lesions usually met with in phthisis. In our patient there were not only no ulcers in the intestines, but there was no inflammation or redness present, another additional peculiarity of interest in this case.

Case II.—*Pleurodynia—Necessity of combatting promptly and energetically pleurodynic pains in order to avoid consecutive pleuritic symptoms.*—At No. 21 of the Salle St. Agnes, is a young man, of twenty-three years of age, of a strong constitution, and a tailor by trade. In the midst of excellent health he was suddenly seized, about ten days since, with violent pain just below the right nipple, so severe as to oblige him to relinquish his work. On the next day (Dec. 1.) in spite of the persistence of these pains he resumed his occupation, but was unable to support for anytime the fatigue of work, and having in vain struggled against them for three or four days, he finally decided on entering the hospital.

Questioned as to his having been exposed to the influence of cold; if he inhabited a cold, damp room, or if by accident he had slept with his windows open; he has answered in the negative on all points. He assures us, moreover, that he has made no effort, that he has not overworked himself, that he has committed no excess in diet, and that he has received no blow on the chest. It was important to insist on all these details, in order that we might be enlightened with regard to the etiology of the disease which presented itself in the form and with the appearance of rheumatism. The patient, besides, has had no fever. There is some diminution of appetite, and some constipation; this latter symptom is due perhaps to the decrease in diet. The patient suffered from no chill at the debut; the heat of the skin is natural; the pulse is about sixty; no headache; nothing anormal in the digestive apparatus. The pain in the side is fixed upon the point just indicated; it is increased by the slightest movement, as walking, coughing, blowing the nose, &c. Walking is followed by some degree of dyspnœa, but it seems to be caused by the fear of increasing the pain, in indulging in too deep respiration. Besides neither percussion or auscultation reveal anything anormal in the respiratory sound, the seat of pain, except a slight diminution in the force, which ought to be attributed to the circumstance which I have just noticed. When the skin is pinched, when you raise it up, or when you press upon the seat of pain, there is increase of pain; this excess of sensibility is not in the skin itself, but in the subjacent tissue. It is easy by these symptoms to recognise a rheumatismal affection of the pectoral muscles of the right side, or in other words a pleurodynia.

At first sight you may regard this affection as but of small importance, and of little gravity, more particularly as the patient states that he has never previously suffered from any rheumatismal symptoms, and that his family has been equally exempt. However, in spite of the apparent benignity, rheumatismal pains of the chest merit all our attention. If they persist for any time, they are almost invariably followed by pleurisy, with effusion in the side corresponding with the pain. Sometimes the effusion forms, by degrees almost insensibly, without any evident precursory symptoms, and without the knowledge either of the patient, and often of the physician. It very often happens that the medical attendant having ausculted a patient at the commencement of pleurodynic symptoms without finding anything anormal, and not thinking to repeat his exploration, is surprised to find some time subsequently his patient suffering] from dyspnœa, with impeded respiration, and, on examining him again, to discover a considerable pleuritic effusion. This should make you frequently examine the chest of patients thus affected, in order to be averted of the consecutive lesions that may follow.

The production of serous effusions in the pleura from rheumatism of the thorax, bears a strong analogy to the serous effusions in the cynovial membranes and the articulations, in consequence of articular rheumatism, as in the knee and elbow, for example. These two kinds of effusion are governed, by all appearances, by the same law. Do not therefore neglect pleurodynic pains, especially when they last any time; treat them on the contrary with care, promptly, and with energy, according to the state of the case.

There is another kind of pleurodynia accompanied with fever. In this case we are sure of consecutive pleurisy and effusion. Then, above all, it is important not to be beguiled into dangerous inaction. You must act energetically, bleed largely and repeatedly, according to the strength of your patient, and the intensity of the symptoms, for, I repeat, without this you expose your patient to dangerous accidents.

In the present case, although there is no fever, yet as the pain is so acute, and as it shows a disposition to increase rather than diminish, I shall apply a number of scarified cups to the right side, over the seat of pain. Generally this treatment succeeds very well; should it not afford relief, we shall have recourse to a blister, and other suitable means.

*Paris, Dec.* 1842.

